# Gel immersion endoscopic mucosal resection of a duodenal adenoma with gastric phenotype

**DOI:** 10.1055/a-2325-2143

**Published:** 2024-06-05

**Authors:** Kensuke Suzuki, Daisuke Kikuchi, Satoshi Yamashita, Kei Kono, Yutaka Takazawa

**Affiliations:** 1 559349 Gastroenterology, Toranomon Hospital Branch, Kawasaki, Japan; 2 13600 Gastroenterology, Toranomon Hospital, Tokyo, Japan; 3 13600 Pathology, Toranomon Hospital, Tokyo, Japan

Endoscopic mucosal resection (EMR) is the most common endoscopic intervention for duodenal neoplasms. The literature describes various options, including cold snare polypectomy, underwater EMR (UEMR), and endoscopic submucosal dissection (ESD) 1 2 . The gel immersion EMR method has been developed in recent years to achieve safe and easy en bloc resection 3 4 . Here, we report the en bloc resection of an elevated lesion with a prominent depression in the duodenal bulb using the gel immersion EMR technique.

An elderly man (age >80 years) was found to have a 20-mm elevated lesion in the duodenal bulb ( Fig. 1 ). Biopsy of this revealed a duodenal adenoma with gastric phenotype. With the use of narrow-band imaging (NBI) magnification, granular mucosal micropatterns of varying sizes were observed ( Fig. 2 ). Endoscopic ultrasound (EUS) revealed that the duodenal muscularis propria maintained a circular shape and was not aligned with the fold involutions ( Fig. 3 ). We performed gel immersion EMR ( Video 1 ). The lesion was resected en bloc after securely entrapping the normal mucosa surrounding the lesion within the 15-mm snare 5 ( Fig. 4 **a** ). No perforation or any other muscular layer damage was observed, and the EMR ulcer was closed with clips. Pathologic findings confirmed a 17×14-mm duodenal adenoma with gastric phenotype, with negative margins ( Fig. 4 **b** ).

**Fig. 1:**
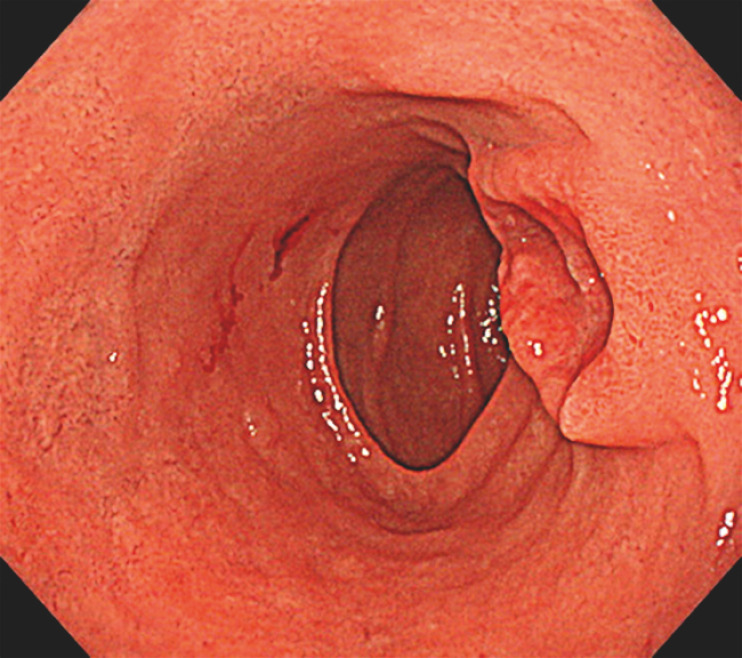
White-light endoscopic image showing a 20-mm elevated lesion with a prominent depression in the center at the duodenal bulb.

**Fig. 2:**
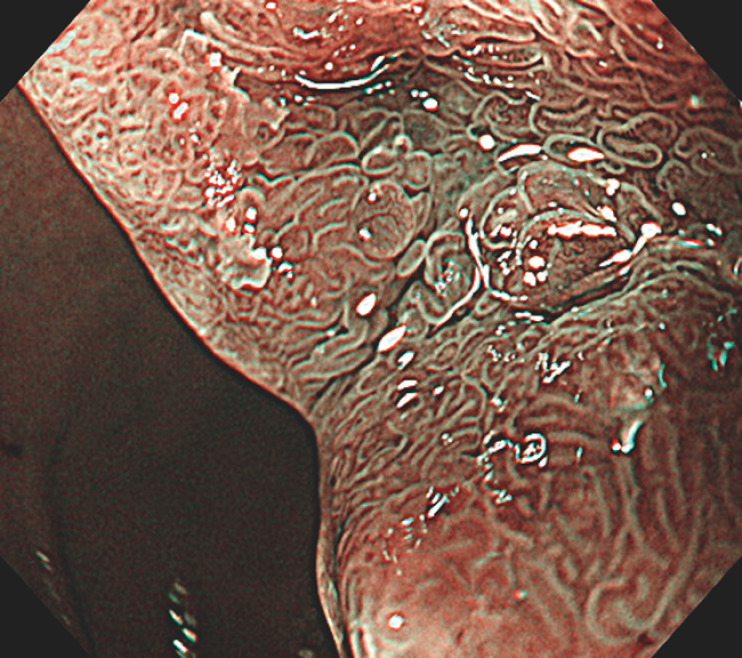
Narrow-band imaging magnification image of the lesion, which was diagnosed as being a duodenal adenoma with gastric phenotype.

**Fig. 3:**
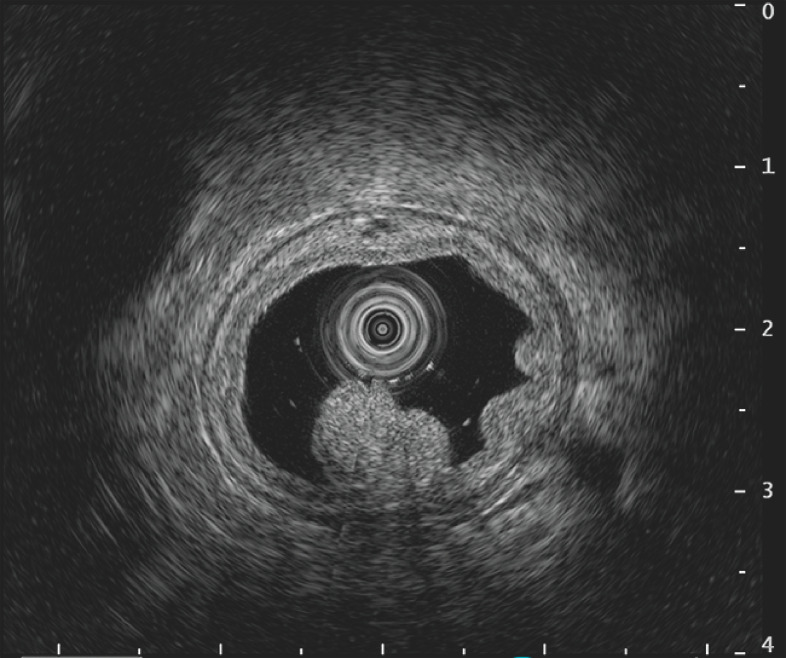
Endoscopic ultrasound image showing that the duodenal muscularis propria was preserved in a circular shape and was not adhering to the involutions of the folds.

Gel immersion endoscopic mucosal resection is performed for a duodenal adenoma; visualization of the lesion was improved with the use of Viscoclear.Video 1

**Fig. 4:**
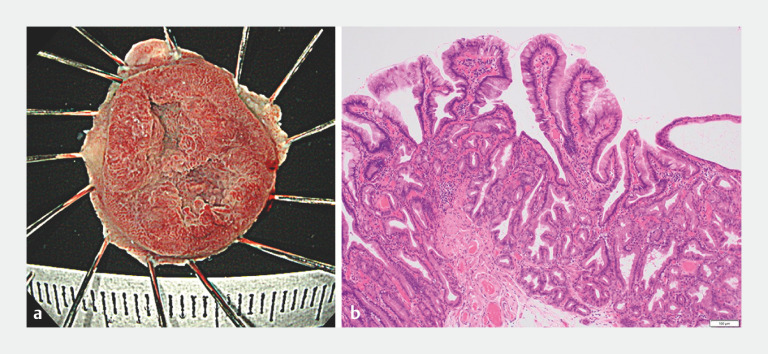
The resected specimen: **a** on macroscopic view, showing the lesion was resected completely en bloc; **b** on histopathologic microscopic view, showing a 17×14-mm duodenal adenoma with gastric phenotype, with negative margins.

Gel immersion EMR has been associated with a higher R0 resection rate than UEMR 3 . Similarly to UEMR, the duodenal muscularis propria is not pulled to the lesion side when water is stored and it is therefore considered a safe treatment. Likewise, in this case, EUS was used to confirm the course of the duodenal muscularis propria, and gel immersion EMR was performed. A local injection may not obtain a protuberance in the center of a lesion that has a prominent depression, such as in this case. Gel immersion EMR may therefore be useful for such lesions.

Endoscopy_UCTN_Code_TTT_1AO_2AG_3AC

## Notes

Endoscopy E-Videos https://eref.thieme.de/e-videos
*E-Videos* is an open access online section of the journal *Endoscopy* , reporting on interesting cases and new techniques in gastroenterological endoscopy. All papers include a high-quality video and are published with a Creative Commons CC-BY license. Endoscopy E-Videos qualify for HINARI discounts and waivers and eligibility is automatically checked during the submission process. We grant 100% waivers to articles whose corresponding authors are based in Group A countries and 50% waivers to those who are based in Group B countries as classified by Research4Life (see: https://www.research4life.org/access/eligibility/ ). This section has its own submission website at https://mc.manuscriptcentral.com/e-videos .
